# Knockdown of Pyruvate Kinase M2 Inhibits Cell Proliferation, Metabolism, and Migration in Renal Cell Carcinoma

**DOI:** 10.3390/ijms20225622

**Published:** 2019-11-10

**Authors:** Prasanta Dey, Ji Yeon Son, Amit Kundu, Kyeong Seok Kim, Yura Lee, Kyungsil Yoon, Sungpil Yoon, Byung Mu Lee, Ki Taek Nam, Hyung Sik Kim

**Affiliations:** 1 School of Pharmacy, Sungkyunkwan University, Suwon 16419, Korea; deyprasantadey@yahoo.com (P.D.); twiase@naver.com (J.Y.S.); amitjupcl@gmail.com (A.K.); kyeongseok@skku.edu (K.S.K.); syoon88@gmail.com (S.Y.); bmlee@skku.edu (B.M.L.); 2 Severance Biomedical Science Institute, College of Medicine, Yonsei University, Seoul 03722, Korea; yura@yuhs.ac (Y.L.); kitaek@yuhs.ac (K.T.N.); 3 Comparative Biomedicine Research Branch, Division of Translational Science, National Cancer Center, 323 Ilsandong-gu, Goyang-si, Gyeonggi-do 10408, Korea; kyoon@ncc.re.kr

**Keywords:** pyruvate kinase M2, autophagy, metabolism, migration, invasion

## Abstract

Emerging evidence indicates that the activity of pyruvate kinase M2 (PKM2) isoform is crucial for the survival of tumor cells. However, the molecular mechanism underlying the function of PKM2 in renal cancer is undetermined. Here, we reveal the overexpression of PKM2 in the proximal tubule of renal tumor tissues from 70 cases of patients with renal carcinoma. The functional role of PKM2 in human renal cancer cells following small-interfering RNA-mediated *PKM2* knockdown, which retarded 786-O cell growth was examined. Targeting PKM2 affected the protein kinase B (AKT)/mechanistic target of the rapamycin 1 (mTOR) pathway, and downregulated the expression of glycolytic enzymes, including lactate dehydrogenase A and glucose transporter-1, and other downstream signaling key proteins. PKM2 knockdown changed glycolytic metabolism, mitochondrial function, adenosine triphosphate (ATP) level, and intracellular metabolite formation and significantly reduced 786-O cell migration and invasion. Acridine orange and monodansylcadaverine staining, immunocytochemistry, and immunoblotting analyses revealed the induction of autophagy in renal cancer cells following PKM2 knockdown. This is the first study to indicate PKM2/AKT/mTOR as an important regulatory axis mediating the changes in the metabolism of renal cancer cells.

## 1. Introduction

Renal cell carcinoma (RCC) is the most common type of kidney cancer, and approximately 64,000 new cases were diagnosed in the United States of America in 2017 [1,2]. Among the main histological subtypes of RCC, clear cell renal cell carcinoma (ccRCC) is deemed as the most hostile, responsible for 70–80% of all RCC cases [3,4]. The prognosis of patients with ccRCC exhibiting distant metastasis is poor, with a 5-year survival rate of nearly 12% [5], and nearly 20–40% of patients suffer from distant metastasis [6].

The resistance of cancer cells to chemotherapy and radiation therapy demands the development of new therapeutic strategies [7]. Hence, it is essential to identify the molecular mechanisms underlying ccRCC to prevent metastasis and provide guidance for clinical decision-making and developing novel therapeutic strategies. The development of targeted therapeutics, including multitargeted tyrosine kinase (TK) and mechanistic target of rapamycin 1 (mTOR) inhibitors, has been a major breakthrough in the discovery of interventions against ccRCC [7]. New therapeutic strategies have emerged as effective treatment options against advanced ccRCC through the reprogramming of cancer cell metabolism and use of glycolysis inhibitors [8]. 

In general, cancer cells require a huge blast of energy for growth, division, and survival that is available by the absorption of nutrients, like glucose, and their successive catabolism through a sequences of metabolic reactions including glycolysis and cellular respiration through oxidative phosphorylation [9]. Despite abundant oxygen, cancerous cells achieve a cancer-specific glycolytic scheme called the “Warburg effect,” characterized by the rapid consumption of glucose and its conversion to lactate to meet their high energy demand for survival and proliferation [10,11]. The detailed mechanism underlying this aerobic glycolysis still remains unclear. In recent years, pyruvate kinase M2 (PKM2), the final rate-limiting enzyme of glycolysis, has gained attention. *PKM2* is an alternatively spliced variant of the *PKM* gene that is highly expressed in various cancers and provides selective growth advantages for tumor formation over its counterpart *PKM1* [12,13]. Overexpression of PKM2 results in increased glucose uptake, lactate production, and autophagy inhibition, thereby accelerating oncogenic growth [14].

Aside from its function as a glycolytic enzyme in cancer cells, PKM2 is engaged in various cellular procedures owing to the identification of interacting proteins in the cytoplasm [15,16]. PKM2 interacts with extracellular signal-regulated kinase 1/2 (ERK1/2) and hypoxia-inducible factor-1 (HIF-1) to upregulate the expression of c-Myc and cyclin D1. The consequences include the activation of glycolytic enzymes, including glucose transporter 1 (GLUT1) and lactate dehydrogenase A (LDHA), G1-S phase transition, chromosome segregation, and cell-cycle progression, ultimately promoting tumorigenesis [17]. However, the oncogenic role of PKM2 in RCC has been explored little. 

In the present study, we investigated whether PKM2 promotes the progression of ccRCC tumorigenesis and the mechanism underlying PKM2-mediated regulation of cancer cell metabolism to understand the molecular mechanisms involved in RCC development. Our data clearly demonstrate that PKM2 is overexpressed in RCC tissues as compared with normal renal tissues and that PKM2 knockdown decreases the production of major glycolytic metabolites (pyruvate and lactate). Furthermore, PKM2 knockdown significantly reduces cell viability and induces autophagy via the protein kinase B (AKT)/mTOR pathway. Our findings clearly indicate that PKM2 regulates the viability of 786-O cells and that targeting PKM2 could reduce the Warburg effect and serve as a potential therapeutic strategy for RCC.

## 2. Results

### 2.1. Identification of PKM2 Expression in RCC

**Figure 1:**
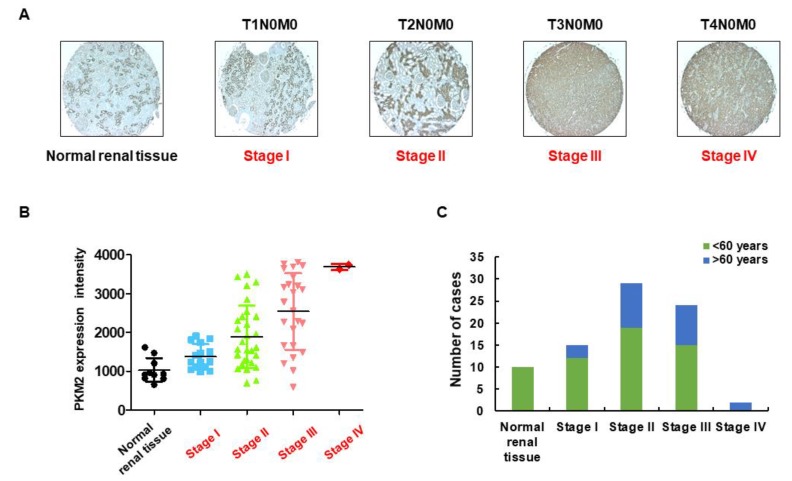
Expression level of pyruvate kinase M2 (PKM2). (**A**) PKM2 protein was immunostained with a specific antibody in normal human kidney tissue and kidney cancer tissue samples and observed under microscopy at 400× magnification. In comparison with normal kidney tissues, kidney cancer tissues exhibited higher expression levels of PKM2. (**B**) Immunoreactive scoring of PKM2 between human kidney cancer tissue samples (at various tumor stages) and normal kidney tissue samples. (**C**) Number of human kidney cancer tissue samples (at various tumor stages) and normal kidney tissue samples.

Studies have revealed overexpression of *PKM2* mRNA in various human cancers, including liver [18], bladder [19], breast [20], lung [21], esophagus [22], gastric, and colorectal [23] cancers. Furthermore, overexpression of PKM2 protein has been associated with different types of human cancers. Here, we performed IHC to investigate the expression of PKM2 protein in a cohort of 70 tissue samples derived from patients with kidney cancer (age, 30–80 years; duplicates per case) and 10 nontumor tissues (age, 14–50 years). As shown in Figure 1A–C, PKM2 protein expression was mainly localized in the nucleus and cytoplasm (stained as brownish granules) and was significantly higher in various kidney cancer tissues (depending on the tumor stage) than in normal tissues. A summary of the clinicopathological features of all tissues is indicated in the Supplementary Materials (Table S1). We also compared the basal level of PKM1 and PKM2 expression in different cancer cell lines and found that metastatic renal cancer 786-O cells exhibited relatively stronger expression of PKM2 than other cancer cell lines [24].

### 2.2. PKM2 Knockdown Inhibits Tumor Progression of 786-O Cells

**Figure 2:**
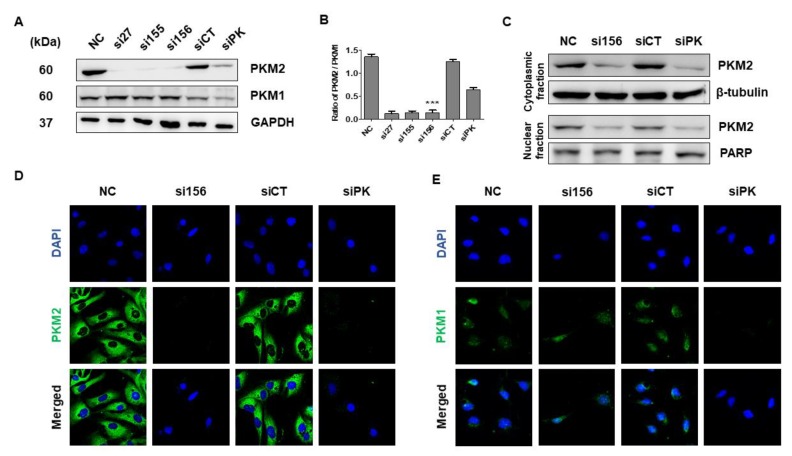
Effect of small interfering RNA (siRNA) transfection on PKM2 and PKM1 expression. (**A**) Expression level of PKM2 was drastically reduced in cells transfected with indicated *PKM2* siRNAs. No changes were observed in PKM1 expression level after *PKM2* siRNA transfection. PKM1 expression level was reduced after transfection with siPK. Glyceraldehyde 3-phosphate dehydrogenase (GAPDH) was used as internal loading control. (**B**) Statistical quantification of immunoblot bands of PKM2/PKM1. One-way ANOVA was used to compare means of various groups. Alterations between mean values were considered significant at *p* < 0.05 using Tukey’s multiple comparison test; *** *p* < 0.001 as compared with normal control cells. (**C**) Western blot analysis of expression level of PKM2 in cytoplasmic and nuclear fractions of 786-O cells after transfection with indicated siRNAs. β-Tubulin and poly (ADP-ribose) polymerase (PARP) were used as internal loading controls for cytoplasmic and nuclear fractions, respectively. (**D**,**E**) Immunofluorescence analysis of PKM1 and PKM2 expression after siRNA transfection at 400× magnification. *PKM2* siRNA transfection drastically reduced PKM2 expression in the cytoplasm and nucleus, as confirmed by the absence of PKM2 green signal compared with normal and negative control cells. Positive control siPK also reduced PKM2 expression. PKM1 was expressed in normal and siCT- and si156-transfected 786-O cells. Expression of PKM1 protein was reduced in siPK-transfected cells as compared with other groups. 4,6-diamidino-2-phenylindole dihydrochloride (DAPI) was used for nuclear staining, and Alexa Fluor 488-conjugated goat anti-rabbit antibody was used to detect PKM2 and PKM1 expression. si27, si155, and si156: experimental siRNAs targeting *PKM2* mRNA; siPK: commercially available siRNA targeting both *PKM1* and *PKM2* mRNAs (positive control); and siCT (siControl): siRNA targeting the firefly luciferase gene (negative control).

To confirm the most effective siRNA against *PKM2* and investigate the role of PKM2 in tumor progression, 786-O cells were transfected with the indicated *PKM2* siRNAs. As shown in Figure 2, si156 treatment (100 nM for 72 h) significantly decreased PKM2 protein expression, without any effect on PKM1 expression level, as compared with cells from normal and negative control groups (Figure 2A,B). PKM2 expression was significantly downregulated in the cytoplasmic and nuclear extracts following si156 treatment, consistent with the above observations (Figure 2C). To determine the siRNA that exhibited a potent PKM2-silencing effect, 786-O cells were transfected under various conditions. As a result, we found a robust decline in PKM2 protein expression in 786-O cells transfected with 100 nM siRNA for 72 h, without any effect on PKM1 expression (Figure S1). The significant knockdown of PKM2 expression mediated by si156 in 786-O cells was further confirmed with immunocytochemistry (Figure 2D). The expression of both PKM1 and PKM2 was downregulated in siPK-transfected cells (Figure 2A,E). We also explored the effect of PKM2 downregulation on cell viability, morphology, and growth.

**Figure 3:**
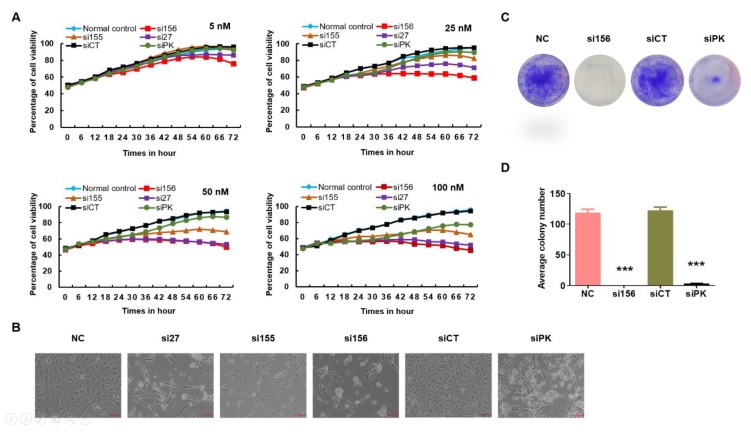
Consequences of PKM2 knockdown to morphology, survival, and colony formation ability of 786-O cells. (**A**) Evaluation of the effect of PKM2 knockdown on viability (determined using IncuCyte ZOOM™ live cell imaging system) of kidney cancer 786-O cells following treatment with indicated siRNAs at concentrations ranging from 5 to 100 nM for 72 h. At a concentration of 100 nM for 72 h, si156 significantly reduced the survival of 786-O cells as compared with other groups. (**B**) Morphology of untransfected and transfected 786-O cells captured at 100× magnification. Morphological changes were prominent after PKM2 knockdown as compared with normal and negative control cells. (**C**) Representative photographs of colony formation assay of 786-O cells transfected with indicated *PKM2* siRNAs in six-well plates captured at 100× magnification. All cell colonies were visually recorded and counted under a light microscope. No differences in colony number were observed between normal and negative control cells as compared with si156-, si155-, and si27-transfected cells. Knockdown of *PKM1* and *PKM2* by siPK reduced the colony number. (**D**) Quantitative analysis of colony number. Representative data of three independent experiments are shown. One-way ANOVA was used to compare means of different groups. Differences between means were considered significant at *p* < 0.05 using Tukey’s multiple comparison test; *** *p* < 0.001 as compared with normal control cells. si27, si155, and si156: experimental siRNAs targeting *PKM2* mRNA; siPK: commercially available siRNA targeting both *PKM1* and *PKM2* mRNAs (positive control); and siCT (siControl): siRNA targeting the firefly luciferase gene (negative control).

**Figure 4:**
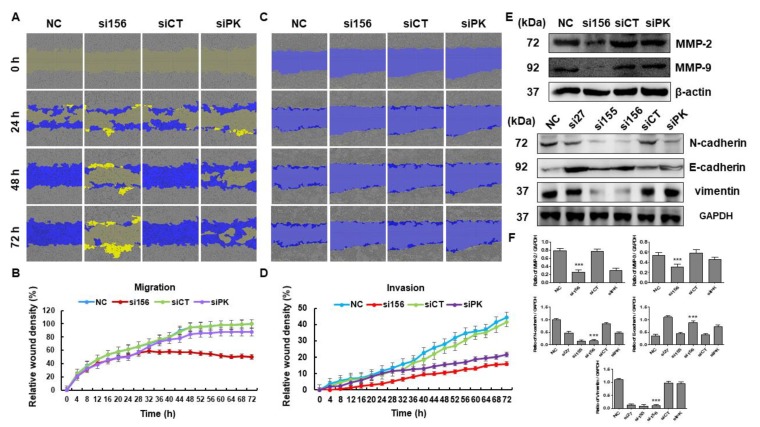
Effects of PKM2 knockdown on migration and invasion of 786-O cells. (**A**) A wound was formed and migration of 786-O cells was measured every day using a phase-contrast microscope for 72 h. (**B**) Mean relative wound density (migration) from three replicate experiments is shown. (**C**) Invasion was assessed in control and PKM2-knockdown cells. (**D**) Mean relative wound density (invasion) from three replicate experiments is shown. (**E**) Cells were harvested and whole cell lysates were examined by Western blotting using antibodies specific for matrix metalloproteinase (MMP)-2, MMP-9, N-cadherin, E-cadherin, and vimentin. GAPDH was used as internal loading control. (**F**) Band intensities were measured and plotted as a bar graph relative to GAPDH level. One-way ANOVA was used to compare means of different groups. Differences between means were considered significant at *p* < 0.05 using Tukey’s multiple comparison test; *** *p* < 0.001 as compared with normal control cells.

In the cell viability assay, si156 transfection reduced the viability of 786-O cells as compared with cells from the negative and normal control groups in a time-dependent manner (Figure 3A and Figure S2). Knockdown of both PKM1 and PKM2 expression resulted in a reduction in the viability of 786-O cells as compared to normal control cells (Figure 3A). Furthermore, si156-mediated knockdown of PKM2 expression not only reduced viability but also induced remarkable morphological changes in 786-O cells (Figure 3B). We monitored the effect of PKM2 downregulation on cell growth. In the colony formation assay, the number of colonies was higher in cells from the normal and negative control groups than in those from the si156 or siPK group, reconfirming the positive effects of PKM2 on 786-O cell growth (Figure 3C,D). We also confirmed that PKM2 knockdown altered the migratory and invasive properties of 786-O cells, as confirmed by the in vitro wound healing assay results. The wound healing course of both control groups was significantly higher than that reported for si156-treated cells (Figure 4A–D). We performed Western blotting for matrix metalloproteinase (MMP)-2/9 and found that PKM2 knockdown altered the expression of these proteins (Figure 4E,F).

Fan et al. reported that during cancer progression and metastasis, polarized epithelial cancer cells converted into contractile and motile mesenchymal cells (known as epithelial–mesenchymal transition, or EMT) [25]. Here, to investigate the role of PKM2 on EMT in kidney cancer cells, we examined the expression level of classical epithelial (E-cadherin) and mesenchymal (N-cadherin and vimentin) cells after PKM2 knockdown. The expression of E-cadherin was downregulated, whereas N-cadherin and vimentin were upregulated in the PKM2-overexpressed normal and negative controls (Figure 4E,F). However, PKM2-knockdown cells showed a decreased level of mesenchymal markers (N-cadherin and vimentin), while a significant upregulation of epithelial marker (E-cadherin) was observed (Figure 4E,F). Our results show that PKM2 knockdown may inhibit the EMT in kidney cancer cells.

### 2.3. PKM2 Knockdown Induces Autophagy

**Figure 5:**
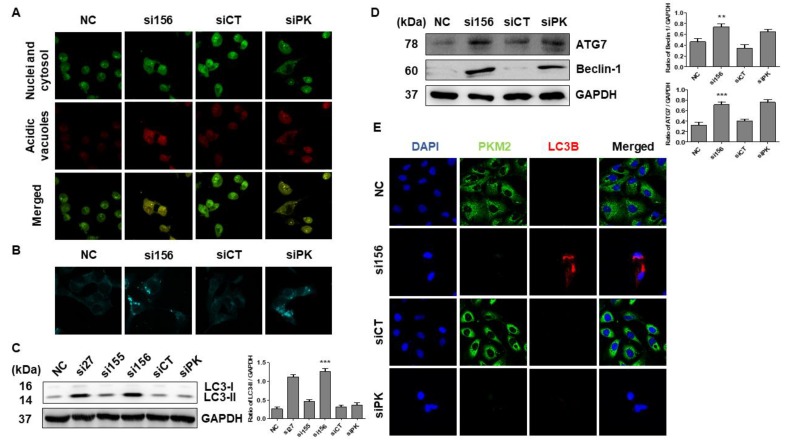
Evaluation of induction of autophagy after PKM2 expression knockdown. (**A**) 786-O cells were transfected with specified siRNAs and stained with acridine orange (1 μg/mL). Formation of acidic vesicular organelles (AVOs) was observed under a confocal microscope at 400× magnification. Fluorescent green staining in cytoplasm and nucleus and fluorescent bright red or orange-red staining of autophagic vacuoles were observed. (**B**) Analysis of autolysosome formation through MDC staining. Cells were transfected, stained with MDC (50 μM), and examined using confocal microscopy. Scale bars indicate 50 μm. (**C**,**D**) Western blot analysis of autophagic proteins in 786-O cells. Immunoblotting was performed using whole-cell lysates, and GAPDH was used as internal loading control. Representative blots are shown. Ratios of LC3-II/GAPDH and beclin 1/GAPDH were measured and depicted as bar graphs. One-way ANOVA was used to compare means of different groups. Differences between means were considered significant at *p* < 0.05 using Tukey’s multiple comparison test; ** *p* < 0.01, and *** *p* < 0.001 as compared with normal control cells. (**E**) Immunofluorescence analysis was performed to evaluate expression of light chain 3B (LC3B) after siRNA transfection and observed under a confocal microscope at 400× magnification. Higher LC3B expression (indicated by red fluorescence) was observed in si156-transfected cells than in siPK-transfected, normal control, and negative control cells. DAPI was used for nuclear staining. Alexa Fluor 488-conjugated goat anti-rabbit antibody and goat anti-mouse IgG (H+L) cross-adsorbed secondary antibody Rhodamine Red-X were used to detect PKM2 and LC3B, respectively.

**Figure 6:**
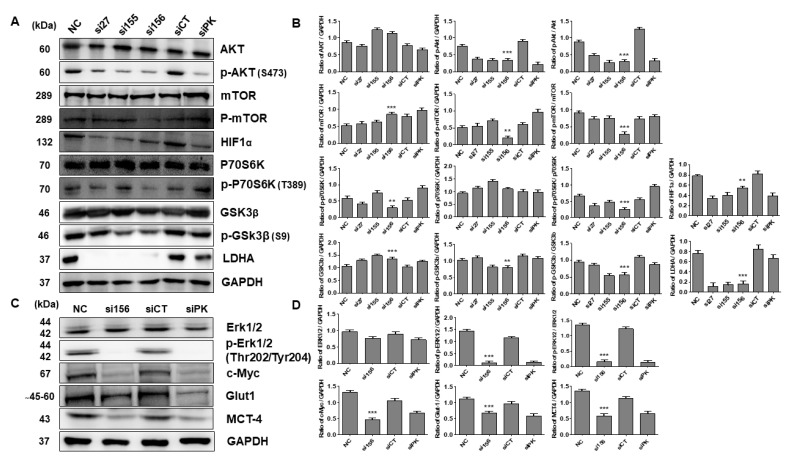
Expression patterns of proteins involved in signaling pathways after PKM2 knockdown by siRNAs. (**A**) Effect of PKM2 knockdown on cancer signaling pathways in 786-O cells. Cells were transfected with siRNAs and immunoblotting was performed to evaluate expression levels of signaling pathway proteins. Representative blots are shown. (**B**) Band intensities were measured and represented as bar graphs relative to GAPDH level. Ratios of p-AKT/AKT, p-mTOR/mTOR, p-GSK3β/GSK3β, and p-p70S6K/p70S6K were measured and depicted as bar graphs. One-way ANOVA was used to compare means of different groups. Differences between means were considered significant at *p* < 0.05 using Tukey’s multiple comparison test; ** *p* < 0.01, and *** *p* < 0.001 as compared with normal control cells. (**C**) Effect of PKM2 knockdown on expression levels of glycolytic signaling proteins. PKM2 knockdown downregulated expression levels of various glycolytic proteins, thereby reducing glycolysis in *PKM2* siRNA-transfected 786-O cells. Representative blots are shown. (**D**) Band intensities were measured and illustrated as bar graphs relative to GAPDH level. Ratio of p-ERK1/2/ERK1/2 was measured and depicted as a bar graph. One-way ANOVA was used to compare means of different groups. Differences between means were considered significant at *p* < 0.05 using Tukey’s multiple comparison test; ** *p* < 0.01, and *** *p* < 0.001 as compared with normal control cells.

Overexpression of PKM2 is known to inhibit autophagy induction through the activation of mTORC1 (a master regulator of autophagy) in various cancer types [14,23,26]. To investigate whether PKM2 exerts any role in autophagy of 786-O cells, we performed acridine orange and MDC staining and found that the si156-mediated PKM2 expression knockdown significantly induced acidic and autophagic vacuole formation, as confirmed by the staining results with two acidotropic dyes, acridine orange and MDC, respectively (Figure 5A,B). We also performed Western blot analysis for autophagy-related proteins and found that PKM2 knockdown upregulated beclin 1 and ATG7 expression and LC3 I/II conversion (Figure 5C,D). Treatment with an autophagy inhibitor, 3-MA (2 mM), for 24 h significantly reversed the expression levels of these proteins (ATG7 and LC3 I/II) (Figure S3). Expression of the autophagy marker light chain 3 B (LC3B) was detected in si156-mediated PKM2 knockdown examined by ICC, consistent with the results of immunoblotting (Figure 5E). To elucidate the regulatory pathways implicated in the effect of PKM2 knockdown on RCC cell metabolism, we examined the AMP-activated protein kinase (AMPK) signaling pathway. AMPK is an important regulator of glucose and lipid metabolism. Therefore, we evaluated the association between the role of the AKT/mTOR signaling pathway and autophagy regulation in 786-O cells. As a result, we found that si156-mediated PKM2 knockdown significantly downregulated the expression of p-AKT, p-mTOR, and other related proteins (HIF-1α, c-Myc, p-P70s6K, p-glycogen synthase kinase 3 beta (GSK3β), p-ERK1/2, and Glut-1) without affecting the corresponding total protein levels (Figure 6A–D). Treatment with 3-MA (2 mM) for 24 h further reduced the expression level of phosphor-AKT protein (Figure S3). These results clearly indicate that the AKT/mTOR signaling pathway may play a crucial role in PKM2-induced kidney cancer autophagy.

### 2.4. PKM2 Knockdown Impairs Glycolysis, Glycolytic Intermediates, and Mitochondrial Activity

**Figure 7:**
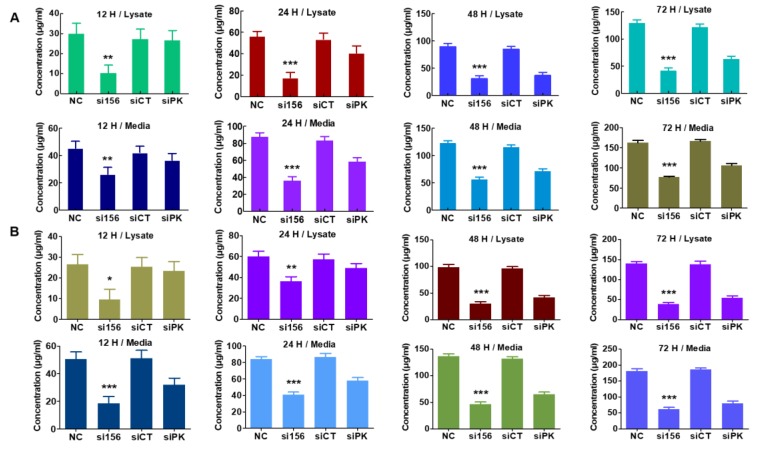
Effect of PKM2 expression knockdown on glycolytic metabolite levels in 786-O cells. (**A**) Quantitative level of pyruvate in lysates and media. PKM2 expression knockdown by si156 significantly reduced pyruvate level in lysates and media of PKM2 siRNA-transfected cells as compared with control cells after 72 h. (**B**) Quantitative level of lactate in lysates and media. si156 considerably reduced lactate level in lysates and media of 786-O cells as compared with control cells. One-way ANOVA was used to compare means of different groups. Differences between means were considered significant at *p* < 0.05 using Tukey’s multiple comparison test; * *p* < 0.05, ** *p* < 0.01, and *** *p* < 0.001 as compared with normal control cells.

**Figure 8:**
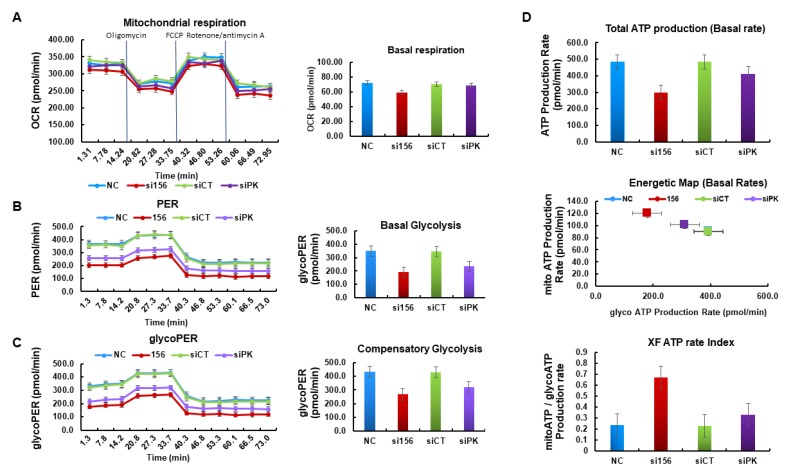
Pyruvate kinase M2 isoform (PKM2) silencing attenuates glycolysis in 786-O cells. (**A**) No significant changes were observed in oxygen consumption rate (OCR) after PKM2 expression knockdown. PKM2 knockdown also inhibited the basal respiration. (**B**) Proton efflux rate (PER) and basal glycolysis decreased after PKM2 expression knockdown by si156. (**C**) Proton efflux rate by glycolysis (glycoPER) and compensatory glycolysis decreased after PKM2 expression knockdown. (**D**) PKM2 knockdown significantly decreased total ATP production rate but increased the rate of mitoATP/glycoATP production.

To understand the mechanism underlying the tumorigenic functions of PKM2, we examined whether PKM2 knockdown affects aerobic glycolysis. PKM2 silencing in 786-O cells attenuated glucose uptake (reduced Glut-1 expression, as shown in Figure 6C,D) and pyruvate and lactate production in cell lysates and media as compared with cells from normal and negative control groups (Figure 7A,B). As seen with immunoblotting, PKM2 expression knockdown significantly downregulated the protein levels of LDHA and MCT4 (Figure 6A–D). Therefore, we determined the concentrations of lactate and pyruvate in the cell media and intact cell lysates to investigate whether PKM2 knockdown changes the intermediate concentration. Knockdown of PKM2 expression in 786-O cells significantly decreased the concentration of pyruvate and lactate; however, treatment with siCT failed to significantly reduce the concentration of lactate and pyruvate (Figure 7A,B). Considering the effects of these enzymes on glycolysis, it seems that the concentration changes in these intermediates may be associated with changes in glycolysis. PKM2 knockdown had a little inhibitory effect on oxygen consumption rate (OCR) (Figure 8A) and basal respiration, indicating that their energy may be mainly generated by mitochondrial oxidative phosphorylation. On the other hand, PKM2 knockdown in 786-O cells resulted in a decrease in total proton efflux rate (PER) and glycolytic proton efflux rate (glycoPER; Figure 8B,C) and caused a decline in glycolysis under basal conditions and compensatory glycolysis, an indicator of decreased aerobic glycolytic rates. 

We observed a significant decrease in ATP production in PKM2-knockdown cells as compared with normal and negative control cells (Figure 8D). This result demonstrates that PKM2 may favor energy production through the Warburg effect. Downregulation of c-Myc and HIF-1α (another regulator of aerobic glycolysis) protein expression levels also supports the above points and suggests that PKM2 may play an important role in the regulation of aerobic glycolysis in human kidney cancer 786-O cells.

## 3. Discussion

RCC, a common cancer arising from the renal tubular epithelial cells, encompasses 85% of all primary renal neoplasms. There has been a steady rise in the incidence and mortality rate of RCC [27]. Several clinical trials demand the development of newer therapeutic approaches for RCC [28,29,30]. The immune system plays a vital role in the recognition and subsequent rejection of several types of cancer [31]. Unfortunately, the immune system is not always adequate in attacking and treating metastatic renal cancer. Thus, identifying a new potential target for selective therapeutic treatment is very important. Metabolic reprogramming of cancer cells is considered as a strategy to combat metastatic cancer and may allow eradication of cancer cells that escape different stress factors imposed by the immune system as well as various drugs. Several drugs that inhibit cancer cell metabolism are effective against various cancers [32,33]. However, only a few studies have explored the relationship between metabolic reprogramming and RCC.

Previous studies have demonstrated that in spite of the availability of sufficient oxygen, cancer cells enhance their glycolytic pathway and use more glucose to synthesize glycolytic intermediates for maintaining their cellular growth, division, and survival. As a result, several inhibitors of glycolytic enzymes have been investigated for their activity against cancer cells [32,33]. Enhancement in aerobic glycolysis is a characteristic feature of rapidly multiplying cells [11,34,35]. This unique metabolic feature allows these cells to produce biomass to meet their energy demands and regular anabolic processes [35]. Human cancer cell line 786-O similarly displays high aerobic glycolysis, which is positively correlated with tumor progression and poor patient outcomes [36,37,38,39]. PKM2 plays a significant role in maintaining the process of aerobic glycolysis. Therefore, understanding the role of PKM2 in aerobic glycolysis and the survival of cancer cells is relevant to ascertain new therapeutic interventions.

To investigate whether PKM2 is critical for the proliferation and survival of renal cancer cells, we investigated PKM2 expression levels in TMA using IHC and immunoblot analyses. Here, we demonstrate that PKM2 expression was comparatively higher in renal cancer tissues than in the adjacent tumor or normal kidney tissues. Higher PKM2 expression levels were also observed in 786-O cells than in other types of cancer cell lines [24]. Based on these results, we used siRNA for PKM2 expression knockdown in 786-O renal cancer cells. The proliferation was significantly inhibited in *PKM2* siRNA-treated cells as compared with wild-type cells. Therefore, we found that the expression of PKM2 in RCC cells was inversely correlated with cell proliferation and survival, suggestive of the relationship between PKM2 overexpression and renal cancer cell proliferation. Accumulating evidence has demonstrated the function of PKM2 in the proliferation of various cancer cells [16]; however, to the best of our knowledge, the relationship between PKM2 and renal cancer cell 786-O remains unclear. In this direction, we investigated the consequences of PKM2 knockdown to the mTOR pathway in renal cancer cells.

mTOR is the most typically activated downstream protein of the phosphoinositide 3-kinase (PI3K)/AKT pathway in response to the signaling of growth factors and is downregulated in a wide spectrum of human cancers. The PI3K/AKT/mTOR pathway is often the front-line target of choice for therapy for patients with metastatic RCC [40]. The potential clinical significance of the PI3K/AKT pathway has been established in numerous studies that confirm the negative prognostic role of low PTEN/high p-AKT/high PI3K expression in patients with RCC. The AKT/mTOR pathway is well characterized and identified to play a pathogenic role in cell proliferation, autophagy maintenance, and tumor development [41]. AKT is a master regulator of cell survival under stress conditions [42], whereas mTOR regulates the synthesis of important proteins and intake and expression of growth factors [43]. The downstream effector mTOR (P70s6K) regulates cell cycle progression, metabolism, and growth [44]. This AKT/mTOR pathway was reported to be frequently activated in RCC and is directly linked to RCC survival and progression [45,46,47,48]. Overexpression of PKM2 results in activation of mTORC1 signaling following phosphorylation of AKT1S1 (an inhibitor of mTORC1), resulting in inhibition of autophagy [14]. Our results indicate that PKM2 knockdown could attenuate the phosphorylation of AKT and subsequently downregulate mTOR phosphorylation and induce autophagy.

We confirmed the deposition of autophagosomes and autophagolysosomes with acridine orange and MDC staining, respectively. In addition, potent expression of LC3B was observed in PKM2-knockdown cells by immunocytochemistry and immunoblot analysis. Furthermore, the expression of other autophagy-specific proteins was upregulated after PKM2 knockdown. The roles of 3-MA and knockdown of PKM2 in autophagy are contradictory. It has been reported that 3-MA can directly inhibit class III PI3K, but also inhibit class I and II PI3K, which ultimately downregulate Akt phosphorylation [49]. After 3-MA (2 mM) treatment for 24 h, expression of P-Akt in PKM2-knockdown cells was further downregulated. On the other hand, upregulation in the expression of autophagy-related proteins after PKM2 knockdown was reversed after 3-MA treatment. The compound 3-MA inhibits autophagy induction through the suppression of class III PI3K activity [49]; however, activation of class I PI3K may lead to induction of the AKT/mTOR signaling pathway, which also inhibits autophagy. Here, we hypothesized that PKM2 knockdown may suppress the activity of class I PI3K without affecting class III PI3K activity, indicating that the AKT/mTOR pathway is crucial for induction of autophagy in PKM2-knockdown cells. Further investigation is warranted to prove the detailed mechanism underlying this hypothesis.

As PKM2 serves as a prime enzyme in the regulation of glycolysis, we investigated the metabolic profile of 786-O cells with XFe96 analyzer. In general, glucose consumption by cells results in net production of lactate and extrusion of a proton into the extracellular medium, thereby causing acidification of the surrounding cellular environment. The rate of proton extrusion in the extracellular medium during glycolysis (glycoPER) was significantly reduced in si156-treated 786-O cells as compared with normal and negative control cells. The cells may also use glucose and other fuels for energy production through mitochondrial respiration. Therefore, mitochondrial-derived CO_2_ is also responsible for extracellular acidification beyond glycolysis. Here, PER was measured immediately after Rot/AA injection (inhibitor of mitochondrial respiration that typically increases glycolysis), but a noticeable inhibition of PER activity was observed in PKM2-knockdown cells as compared to normal and negative control cells. Thus, si156-treated cells showed significant dysregulation in their glycolytic capacity and inhibited mito-acidification after PKM2 knockdown. The addition of 2-deoxy-D-glucose (inhibitor of hexokinase causing a decrease in glycolysis) was intended to ensure extracellular acidification as a result of glycolytic metabolism. Moreover, the significant reduction in basal and compensatory glycolysis observed after PKM2 knockdown reveals the strong association with maintenance of the glycolytic pathway in 786-O cells.

The basal mitochondrial respiration rate (OCR) was measured to monitor any possible changes in the metabolic profile between normal and knockdown cells. PKM2 knockdown had minor effects on OCR; however, the OCR/PER ratio increased in si156-treated cells as compared to normal and negative control cells (data not shown), suggesting a partial metabolic switch to oxidative phosphorylation upon PKM2 inhibition. The cellular ATP production rate is the explanatory measurement to describe cellular metabolism. Therefore, the regulation of cellular metabolism is an important feature that allows cells to change their ATP production rate based on ATP demand, thereby adjusting their ATP level. In mammalian cells, glycolysis and oxidative phosphorylation are the two primary metabolic pathways responsible for ATP production. In 786-O cells, total ATP production was inhibited after PKM2 knockdown. In particular, PKM2 knockdown inhibited the rate of glycolysis and resulted in the blockade of ATP production by glycolysis. As a partial metabolic shift was observed in PKM2-knockdown cells as compared with normal and negative control cells, the ATP production (from oxidative phosphorylation in the mitochondria) was slightly increased in PKM2-knockdown cells. We measured the ATP rate index to detect any changes and/or differences in the metabolic phenotype. This valuable matrix increased in si156-treated cells as compared with control cells. Thus, the cells become more oxidative or less glycolytic after PKM2 knockdown. High-performance liquid chromatography (HPLC) analysis also revealed that the levels of glycolytic metabolites pyruvate and lactate were significantly decreased in si156-treated cells, suggesting that PKM2 plays an important role in maintaining glycolysis in 786-O cells.

Cancer metastasis requires migration and invasion of cancer cells and is the cause of complications in the systemic treatment of patients with RCC [50]. Degradation of the extracellular matrix is a key parameter involved in the initiation of cancer metastasis [51], and MMPs are crucial proteolytic enzymes that help in this degradation process as well as in metastasis promotion [52]. AKT signaling is known to regulate the expression of MMP-2/9 in several cancer cells and control tumor migration as well as invasion [53,54]. The role of PKM2 in cancer metastasis has been reported earlier, but its underlying mechanism still remains unclear. This EMT is a biological process where the epithelial cells lose their polarity and are transformed into motile mesenchymal cells and promote malignancy. In the present study, si156-transfected 786-O cells lost their migratory and invasive property after 72 h of transfection as compared with negative and normal control cells, reflecting the functional role of PKM2 in tumor migration and invasion. To further understand how PKM2 is involved in the regulation of cell migration and invasion, we examined the expression of related proteins by Western blotting. In comparison with normal and negative control cells, PKM2-knockdown cells showed significant inhibition of the expression of phospho-AKT, MMP-2, and MMP-9. Moreover, the expression of other EMT-relevant proteins was also altered after PKM2 knockdown. As a result, the wound-healing capability of 786-O cells was significantly decreased after knockdown of si156-mediated PKM2 expression. Our results confirm that PKM2 may play a crucial role in tumor metastasis. These observations suggest that PKM2 may play an important role in the migration and invasion of 786-O cells. Further molecular experimentation is needed to uncover more details.

Paracrine signaling can influence cell growth. Seeding more than 50% of cells increases the growth rate rather than lowering it. Colony formation assay reflects 3D cell culture assay where cells grow autonomously on a substrate (also known as anchorage-independent growth). Only cancer cells can grow without a substrate; normal cells cannot. Clonogenic cells can proliferate indefinitely to produce large colonies (containing 50 or more cells) or clones, and clonogenic cell survival assay is a basic tool to evaluate in vitro cellular transformation (or ability of cells to form colonies) [55]. In the colony formation assay, there is no cell–cell communication to protect from chemical hazards because plated cell numbers are lower than cell viability assay, and cells are exposed for long periods. As a result, alteration of glycolysis or other growth or signaling factors makes them more highly sensitized by the 786-O cells to form colonies than in the cell survival assay (where the number of cells is high) after PKM2 knockdown. However, the cells in normal and negative control maintain their ability to form colonies. Knockdown of both PKM1 and PKM2 also inhibited the colony formation ability of 786-O cells.

## 4. Materials and Methods

### 4.1. Chemicals and Reagents

Roswell Park Memorial Institute 1640 medium (RPMI-1640) (cat. no. LM011-01) and penicillin/streptomycin (cat. no. LS202-02) were obtained from Welgene Co. (Gyeongsangbuk-do, South Korea), and fetal bovine serum (FBS) (cat. no. 10099-141), Dulbecco’s phosphate-buffered saline (DPBS) (cat. no. 31600-026), trypsin (cat. no. 25300-054), and reduced serum medium Opti-MEM (cat. no. 31985070) were purchased from Gibco Life Technologies (Carlsbad, CA, USA). Lipofectamine RNAiMAX (cat. no. 13778150) and goat anti-mouse IgG (H+L) cross-adsorbed secondary antibody Rhodamine Red-X (cat. no. R6393) were procured from Invitrogen. NE-PER subcellular fractionation kit (cat. no. 78833) and Pierce bicinchoninic acid (BCA) protein assay reagents A and B (cat. nos. 23228, 1859078) were acquired from Thermo Fisher Scientific (Rockford, USA). Acridine orange solution (cat. no. A8097), monodansylcadaverine (MDC) (cat. no. D4008), acetone (cat. no. 270725), goat serum (cat. no. G9023), Triton X-100 (cat. no. T8787), 4,6-diamidino-2-phenylindole dihydrochloride (DAPI) (cat. no. D9542), and thiamine (cat. no. T1270) were supplied by Sigma-Aldrich (St. Louis, USA). Bovine serum albumin (BSA) (cat. no. BSAS 0.1) and crystal violet (cat. no. C1035) were purchased from Bovogen (Biosesang Inc., Gyeonggi-do, Korea). PRO-PREP protein extraction solution (cat. no. 17081) was purchased from Intron Biotechnology. Immobilon Forte Western HRP substrate (cat. no. WBLUF0100) and polyvinylidene difluoride (PVDF) membrane (cat. no. IPVH00010) were procured from Millipore (Tullagreen, Ireland), and lactic acid (cat. no. L0226) from Tokyo Chemical Industry Co., Ltd (Tokyo, Japan). The primary antibodies for PKM1 (cat. no. D30G6), PKM2 (cat. no. 4053), mTOR (cat. no. 2983), glycogen synthase kinase 3 beta (GSK3β) (cat. no. 12456), phosphorylated GSK3β (p-GSK3β; S9) (cat. no. 5558), LDHA (cat. no. C4B5), GLUT1 (cat. no. 12939), beclin 1 (cat. no. 3495), ATG7 (cat. no. 8558), LC3A/B (cat. no. 12741), LC3B (cat. no. 83506), glyceraldehyde 3-phosphate dehydrogenase (GAPDH; cat. no. 5174), AKT (cat. no. 9272), phosphorylated AKT (p-AKT; S473) (cat. no. 9271), phosphorylated mTOR (p-mTOR; S2448) (cat. no. 2971), ribosomal protein S6 kinase beta-1 (P70S6K) (cat. no. 9202), phosphorylated P70S6K (p-P70S6K; T389) (cat. no. 9206), E-cadherin (cat. no. 3195), N-cadherin (cat. no. 14215), and vimentin (cat. no. 5741) were obtained from Cell Signaling Technology, Inc. (Danvers, MA, USA), while those against c-MYC (cat. no. sc-789), HIF1α (cat. no. SC-13515), MMP-2 (sc-10736), MMP-9 (sc-10737), and MCT-4 (cat. no. SC-376140) were supplied by Santa Cruz Biotechnology, Inc. (Texas, USA). Alexa Fluor 488-conjugated goat anti-rabbit antibody (cat. no. ab150077) was acquired from Abcam (Cambridge, UK), and Seahorse XF Cell Mito Stress Test Kit (cat. no. 103015-100), Seahorse XF Glycolytic Rate Assay Kit (cat. no. 103344-100), and Seahorse XF Real-Time ATP Rate Assay Kit (cat. no. 103592-100) were purchased from Agilent technologies (California, USA). IncuCyte^®^ Cell Migration Kit (cat. no. 4493) was procured from Essen BioScience (Ann Arbor, MI, USA). Matrigel (354234) was purchased from BD Biosciences (San Jose, USA).

### 4.2. Kidney Tumor and Normal Tissues

A tissue microarray (TMA) was constructed from paraffin-embedded tumor specimens. TMA slides (cat. no. KD806) of 10 normal human kidney tissue samples and 70 kidney cancer tissue samples were obtained from US Biomax Inc. (Rockville, MD, USA). Detailed information about the TMA samples is given in Table 1.

### 4.3. Immunohistochemical Analysis

Immunohistochemical (IHC) analysis of human RCC tissues in TMA was performed as previously described [8]. To investigate the expression level of PKM2 in normal human kidney tissue and kidney cancer tissue samples, the TMA sections were exposed to anti-PKM2 antibody. The slides were transferred to a xylene chamber and dipped in alcohol and water, followed by incubation with 3% hydrogen peroxide. The slides were blocked and sequentially incubated with a primary antibody against PKM2 (1:1000) and a horseradish peroxidase (HRP)-conjugated secondary antibody (1:10,000), as per the standard protocol. Immunostained slides were visualized using diaminobenzidine tetrahydrochloride, counterstained with hematoxylin, and subjected to microscopy.

### 4.4. Cell Lines and Cell Culture

Human kidney cancer cell line 786-O was purchased from the American Type Culture Collection, Virginia, USA. The cells were maintained in RPMI-1640 supplemented with 10% heat-inactivated FBS, 100 U/mL penicillin, and 100 μg/mL streptomycin at 37 °C in a humidified atmosphere of 5% CO_2_ and 95% air. The cells at the logarithmic growth phase were collected and used for subsequent investigations.

### 4.5. PKM2 Knockdown

The small-interfering RNA (siRNA) sequences targeting *PKM2* isoform (si27, si155, and si156) were used to knock down the expression of human *PKM2* mRNA, as previously described (Table 2). Cells were transfected with 50 and 100 nM siRNA for 0, 48, and 72 h. Transfections were performed in quadruplicate on two independent occasions using Lipofectamine RNAiMAX reagent, according to the manufacturer’s protocol [56] 

### 4.6. Cell Viability Assay

IncuCyte ZOOM™ live cell imaging system (Essen BioScience, Ann Arbor, MI, USA) was used to determine the viability of 786-O cells. The 786-O cells were seeded and transfected (forward transfection) with PKM2 experimental siRNA (si27, si155, si156), siControl (siRNA targeting the firefly luciferase gene; negative control), siPK (commercially available siRNA targeting both PKM1 and PKM2 mRNAs; positive control) at concentrations ranging from 5 to 100 nM for 72 h. The detailed information of *PKM2* siRNAs is provided in Table 2.

### 4.7. Western Blot Analysis

Western blot analysis was performed as previously described [57]. The 786-O cells were transfected with indicated *PKM2* siRNA at 100 nM concentration for 72 h. Cells were harvested and whole-cell lysate was prepared using PRO-PREP cell lysis buffer. The lysate was subjected to 6–12% sodium dodecyl sulfate (SDS) polyacrylamide gel electrophoresis (PAGE) analysis. After electrophoresis, the separated protein bands were transferred onto PVDF membranes and the membranes were blocked with a blocking buffer. The membranes were incubated with primary and corresponding secondary antibodies. The blots were developed using Immobilon Forte Western HRP substrate.

### 4.8. Immunofluorescence Analysis

Transfected 786-O cells were fixed with acetone and incubated with primary antibodies against PKM2 (1:100) and LC3B (1:400), followed by treatment with Alexa Fluor-conjugated secondary antibodies (1:200) and DAPI (0.1 μg/mL), as per the standard protocols. Cells were examined under a fluorescence microscope (FV10i; Olympus Corp., Tokyo, Japan) at 400× magnification.

### 4.9. Subcellular Fractionation

The nuclear and cytoplasmic fractions of the transfected 786-O cells were extracted using the NE-PER subcellular fractionation kit, according to the manufacturer’s instructions. The proteins were quantified and immunoblotting was performed.

### 4.10. Acridine Orange Staining

Acridine orange staining was carried out as previously described [58]. The 786-O cells were seeded and transfected with the appropriate siRNA after reaching adequate confluency. After transfection, the cells were treated with 1 μg/mL of acridine orange (2.7 μM) in serum-free medium at 37 °C for 15 min. After washing with phosphate-buffered saline (PBS), the formation of acidic vesicular organelles (AVOs) was examined under a fluorescence microscope (FV10i; Olympus Corp., Tokyo, Japan) at 400× magnification. Bright green fluorescence was observed in the cytoplasm and nucleus of the stained cells, whereas AVOs emitted a bright red color.

### 4.11. Monodansylcadaverine Staining

Monodansylcadaverine (MDC) staining was carried out as per the previously mentioned method [58]. After transfection, the formation of autophagolysosomes was detected by incubation of the cells with MDC lysosomotropic autofluorescent compound (50 μM) at 37 °C for 15 min. After incubation, the cells were washed with PBS and immediately examined under a fluorescence microscope (FV10i; Olympus Corp., Tokyo, Japan) at 400× magnification.

### 4.12. Analysis of Glycolysis, Glycolytic Intermediates, and Mitochondrial Activity

The bioenergetic parameters glycolysis, glycolytic intermediates, and mitochondrial activity were monitored with the Seahorse XF96 Flux Analyzer (Seahorse Bioscience), according to the manufacturer’s instructions [59]. The bioenergetic parameters oxygen consumption rate (OCR), proton efflux rate (PER), and ATP release rate were determined using a Seahorse XFe96 Analyzer (Seahorse Bioscience, Billerica, MA, USA) according to the manufacturer’s instructions. Briefly, the medium from the transfected cells was replaced with Agilent Seahorse XF assay medium, and the cells were incubated for 45 min to 1 h in a CO_2_-free 37 °C incubator. The cells were subjected to Seahorse XF Cell Mito Stress Test, XF Glycolytic Rate assay, and XF Real-Time ATP Rate assay. The measurements were recorded at definite time intervals recommended by the manufacturer.

Levels of pyruvate and lactate were quantitatively measured with high-performance liquid chromatography (HPLC) (Gilson, France) [8]. In this study, a liquid chromatography (LC) system comprising a LC-321/322/350 pump (Gilson, France), an autosampler (Gilson-234), and a UV/Vis-151 detector (Gilson) was used. A Synergi Hydro-RP C18 column (250 × 4.6 mm, 4 µm, 80 Å; Phenomenex, USA) preceded by a pre-column (Phenomenex) were used for detection and quantification of samples. The flow rate (0.8 and 0.7 mL/min for lactate and pyruvate, respectively) was maintained in the experimental process. Isocratic mobile phases of water with 0.1% phosphoric acid and water with 20 mM potassium phosphate for lactate and pyruvate, respectively, were used in this experiment. The samples were thoroughly mixed with acetonitrile containing thiamine (internal standard), followed by centrifugation. The extracted supernatants were analyzed with HPLC (LC-321/322/350 pump) at 210 and 220 nm wavelengths for lactate and pyruvate, respectively.

### 4.13. In Vitro Cell Migration and Invasion Assay

For both of these assays, 96-well ImageLock plates (Essen Bioscience, Ann Arbor, MI, USA) were used. Cells were grown to confluence on ImageLock plates and wounds were created with an Essen wound-maker device (Essen Bioscience, MI, USA) according to the manufacturer’s protocol and then transfected. For migration assay, siRNAs were mixed with culture medium, while for invasion assay, siRNAs were mixed with culture medium and Matrigel mixture. The extent of cell migration and invasion was recorded and quantified with the IncuCyte live-cell imager (Essen Bioscience, MI, USA) for up to 72 h.

### 4.14. Colony Formation Assay 

After transfection, the cells were fixed and stained with crystal violet. Colonies were counted and normalized to the number in the control group, as previously described [60,61].

### 4.15. Statistical Analysis

Data are expressed as the mean ± standard deviation (SD). Statistically significant differences between the groups were determined using analysis of variance (ANOVA) followed by Tukey’s multiple comparison test. For all tests, a significance level of 5% (*p* < 0.05) was used. 

## 5. Conclusions

**Figure 9:**
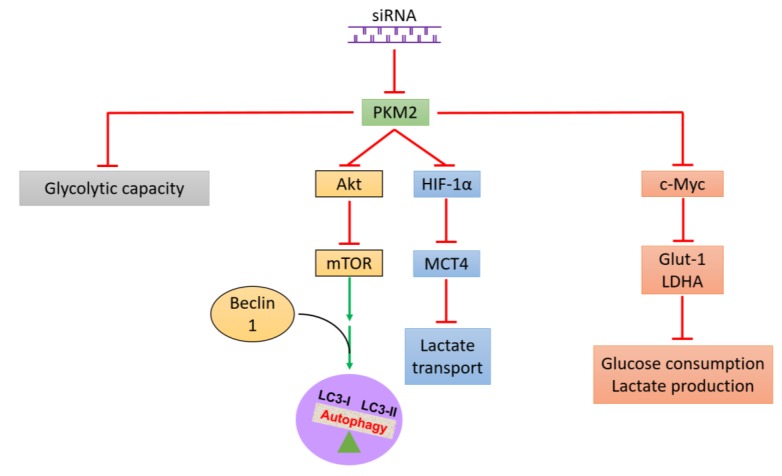
Schematic illustration of consequences of PKM2 expression knockdown to survival and related pathway expression in kidney cancer cells. PKM2 expression knockdown inhibits the AKT/mTOR signaling pathway, thereby activating autophagy and reducing the survival of human kidney cancer cells. Red T-bars represents the inhibition or suppression and green arrows indicates activation.

In this study, we demonstrated that PKM2 is overexpressed in 786-O cell lines as well as in kidney cancer tissues as compared with normal kidney tissues. Therefore, PKM2 expression would be a novel prognostic marker for patients with RCC who receive curative measures. Here, we successfully identified the glycolytic metabolic profile of PKM2 in kidney cancer cells and found that PKM2 inhibition not only suppressed 786-O cell energy metabolism (glycolysis) but also inhibited kidney cancer cell proliferation, survival, migration, and invasion. This is the first report to demonstrate that knockdown of PKM2 expression promotes autophagy, probably mediated by the AKT/mTOR pathway (Figure 9). Our data extend the understanding of the regulatory network of PKM2 in kidney cancer metabolism, cell growth, and development and indicate a potential target for the exploration of therapeutic strategies in kidney cancer.

## Figures and Tables

**Table 1:** Clinicopathological features of experimental samples (human kidney carcinoma tissues and normal kidney tissues).

Tumor Stage and Grade	Variable	No. of Samples	Healthy Subject (%)		Cancer Patient (%)	
			<60	>60	<60	>60
	Patients (Male and Female)	70 Cancer Patients, 10 Healthy Subjects	10 (100%)	-	6 (60%)	4 (40%)
Tumor stage (%)	I	15	-	-	12 (17.14%)	3 (4.29%)
	II	29	-	-	19 (27.14%)	10 (14.29%)
	III	24	-	-	15 (21.43%)	9 (12.86%)
	IV	2	-	-	-	2 (2.86%)
TNM grade	T1N0M0	-	-	-	12 (17.14%)	3 (4.29%)
	T2N0M0	-	-	-	19 (27.14%)	10 (14.29%)
	T3N0M0	-	-	-	7 (10%)	4 (5.71%)
	T3NxM0	-	-	-	1 (1.43%)	1 (1.43%)
	T3aN0M0	-	-	-	5 (7.14%)	4 (5.71%)
	T3bN1M0	-	-	-	1 (1.43%)	-
	T3aNxM0	-	-	-	1 (1.43%)	-
	T4N0M0	-	-	-	-	1 (1.43%)
	T4N1M1	-	-	-	-	1 (1.43%)

TNM grading: T—Primary tumor: Tx—Primary tumor cannot be assessed, T0—No evidence of primary tumor, Tis—Carcinoma in situ; intraepithelial or invasion of lamina propria, T1—Tumor invades submucosa, T2—Tumor invades muscularis propria, T3—Tumor invades through muscularis propria into subserosa or into non-peritonealized pericolic or perirectal tissues., T4—Tumor directly invades other organs or structures and/or perforate visceral peritoneum; N—Regional lymph nodes: Nx—Regional lymph nodes cannot be assessed, N0—No regional lymph node metastasis, N1—Metastasis in one to three regional lymph nodes, N2—Metastasis in four or more regional lymph nodes; M—Distant metastasis, Mx—Distant metastasis cannot be assessed, M0—No distant metastasis, M1—Distant metastasis.

**Table 2:** Sequences of control and PKM2 siRNAs [56].

siRNAs	Sense Stand Sequences	Source
si27	AGGCAGAGGCUGCCAUCUA	Bioneer Corporation
si155	GCCAUAAUCGUCCUCACCA	
si156	CCAUAAUCGUCCUCACCAA	
siCT (negative control siRNA targeting the firefly luciferase gene)	CUUACGCUGAGUACUUCGA	
siPK (positive control siRNA, commercially available, targeting both PKM1 and PKM2 mRNAs)	GGACCUGAGAUCCGAACUG	

## Supplementary Materials

Supplementary materials can be found at https://www.mdpi.com/1422-0067/20/22/5622/s1.

## Abbreviations

PKM2	pyruvate kinase M2
RCC	renal cell carcinoma; LDHA
LDHA	lactate dehydrogenase A
Glut-1	pyruvate kinase M2
siRNA	small-interfering RNA
AKT	protein kinase B
mTOR	mechanistic target of rapamycin 1
ccRCC	clear cell renal cell carcinoma
TK	tyrosine kinase
ERK 1/2	extracellular signal-regulated kinase 1/2
TMA	tissue microarray
HPLC	high-performance liquid chromatography
OCR	oxygen consumption rate
MMP	matrix metalloproteinase
PER	proton efflux rate
glycoPER	glycolytic proton efflux rate
HIF-1	hypoxia-inducible factor-1
LC3B	light chain 3B
AMPK	AMP-activated protein kinase
GSK3β	glycogen synthase kinase 3 beta
PI3K	phosphoinositide 3-kinase
