# Supplementary material for: Knockdown of Pyruvate Kinase M2 Inhibits Cell Proliferation, Metabolism, and Migration in Renal Cell Carcinoma
Source: Int J Mol Sci. 2019 Nov 10;20(22):5622. doi: 10.3390/ijms20225622 (PMC6887957; doi:10.3390/ijms20225622)
Supplement: Supplementary file 1 [file ijms-20-05622-s001.pdf]

Supplementary Figures

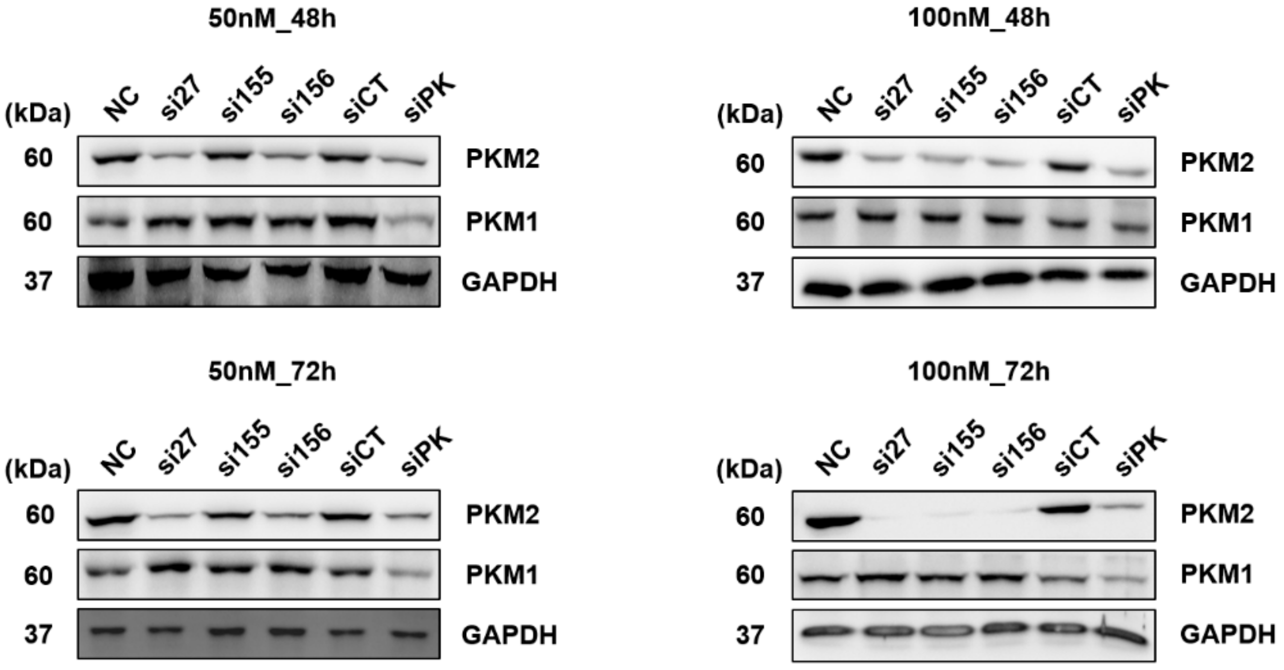

Supplementary Figure S1. Expression profiles of PKM1 and PKM2 proteins after siRNA transfection at various concentrations for different time intervals. GAPDH was used as internal loading control. Representative blots are shown.

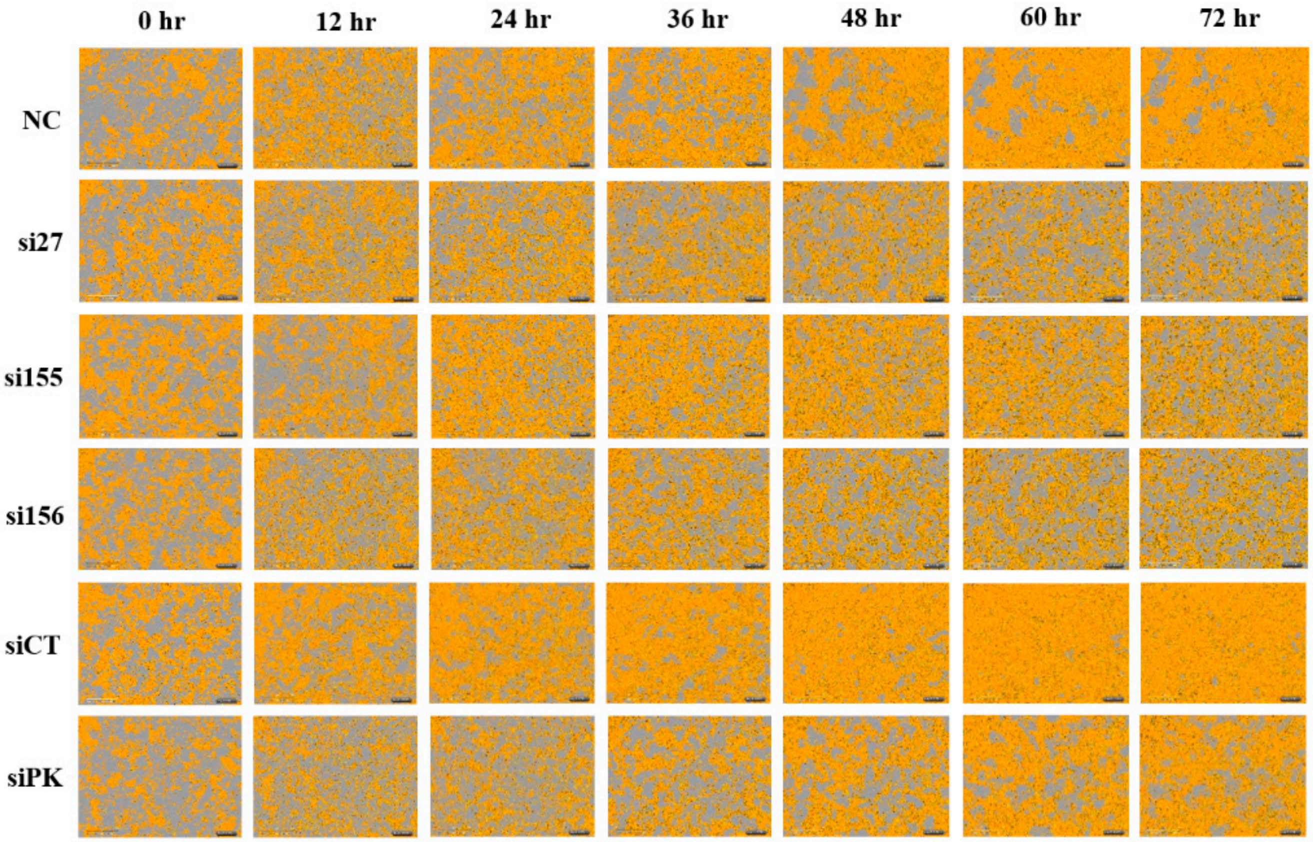

Supplementary Figure S2. Analysis of viability and density of 786-O cells after PKM2 expression knockdown with different siRNAs.

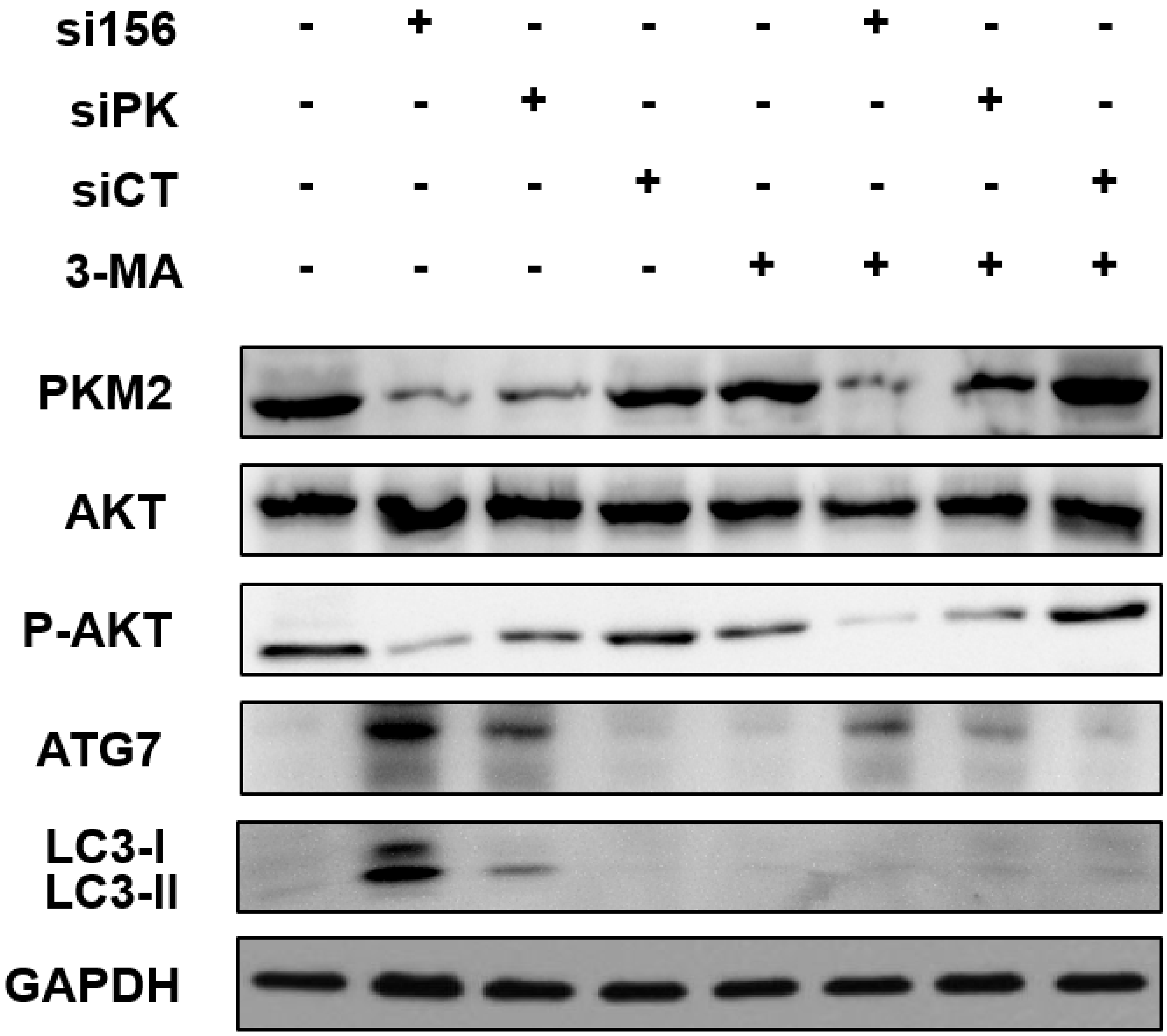

Supplementary Figure S3. PKM2 mediates autophagy via the PI3K/AKT signaling pathway. (**A**) 786-O cells were transfected with si156, siPK, and siCT and treated with 3-MA (2 mM) for 24 h. Cell lysates were prepared and expression levels of PKM2, total AKT, p-AKT, ATG7, and LC3 were analyzed with Western blotting. GAPDH was used as an internal loading control.
